# Optical emissivity dataset of multi-material heterogeneous designs generated with automated figure extraction

**DOI:** 10.1038/s41597-022-01699-3

**Published:** 2022-09-29

**Authors:** Viktoriia Baibakova, Mahmoud Elzouka, Sean Lubner, Ravi Prasher, Anubhav Jain

**Affiliations:** Lawrence Berkeley National Laboratory, 1 Cyclotron Rd, Berkeley, CA 94720 USA

**Keywords:** Optical materials and structures, Materials for optics

## Abstract

Optical device design is typically an iterative optimization process based on a good initial guess from prior reports. Optical properties databases are useful in this process but difficult to compile because their parsing requires finding relevant papers and manually converting graphical emissivity curves to data tables. Here, we present two contributions: one is a dataset of thermal emissivity records with design-related parameters, and the other is a software tool for automated colored curve data extraction from scientific plots. We manually collected 64 papers with 176 figures reporting thermal emissivity and automatically retrieved 153 colored curve data records. The automated figure analysis software pipeline uses Faster R-CNN for axes and legend object detection, EasyOCR for axes numbering recognition, and k-means clustering for colored curve retrieval. Additionally, we manually extracted geometry, materials, and method information from the text to add necessary metadata to each emissivity curve. Finally, we analyzed the dataset to determine the dominant classes of emissivity curves and determine the underlying design parameters leading to a type of emissivity profile.

## Abstract (summary)

**Table:** 

Measurement(s)	optical emissivity • device design
Technology Type(s)	Fourier transform infrared microscopy • text mining

## Background & Summary

Optical device design has impacted many fields, from the pioneering work of Fritts on the selenium solar cell^1^ to the cutting-edge elaboration of nanophotonic intercellular force sensors expanding the conventional microbiology toolkit^2^. Further progress became possible due to materials synthesis^3,4^ and modeling^5,6^ advancements, allowing precise light manipulation over a wide range of wavelengths. Nevertheless, device design optimization remains an iterative process, strongly relying on a good initial guess followed by potentially time-consuming optimization. Modern sources of successful and useful initial designs are databases compiled from digesting the relevant literature, such as Materials Platform for Data Science^7^ and HITRAN^8^.

Optical properties databases should cover as many materials as possible and be up-to-date. There have been several notable endeavors^9–13^ to translate literature into structured databases by parsing the text. However, text-based parsing of data is insufficient for many material properties because much of the needed information is communicated through graphs (e.g., spectral data). The standard method^14^ for converting graphs is manual curve extraction using software such as WebPlotDigitizer^15^, MATLAB GRABIT^16^, DataThief^17^. Manual extraction requires significant user participation (i.e., clicking along the curve). In our experience, it takes approximately 3 minutes to parse a simple graph, which is practical for small tasks but becomes limiting if hundreds of graphs must be extracted. In contrast, existing efforts to automate graph data extraction have a list of drawbacks, such as parsing only continuous curves without sharp picks^18^, requiring the figures to have PDF embedded axes^19^ or having incompatibility issues due to no longer being actively maintained^20^. Therefore, the need exists for a tool for automated curve extraction from plots.

**Fig. 1:**
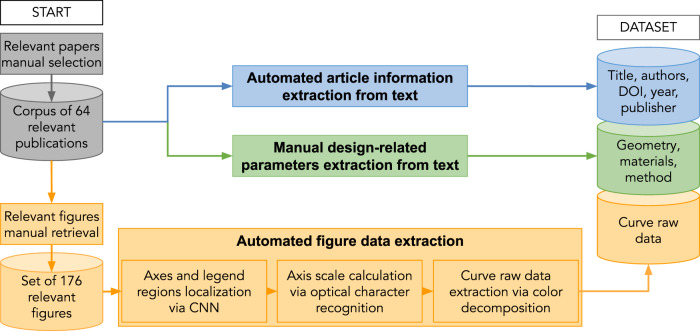
The overall pipeline of data collection and organization into dataset. The data is retrieved from a corpus of 64 manually collected relevant papers (gray). There are three categories of data retrieval: blue - automated extraction of the general article information from text; green - manual extraction of the design-related parameters; and orange - automated curve raw data extraction embedded in semi-automatic figure analysis.

To address the listed issues, we compiled a dataset of thermal emissivity measurements from the optical scientific literature using various image analysis techniques. Figure 1 reviews the overall pipeline, which includes the following steps. First, we manually collected a corpus of 64 relevant publications. From these, we manually retrieved 176 figures containing emissivity-wavelength data relations. We implemented an algorithm for automated curve raw data extraction and automatically obtained data records for 153 curves. Next, we manually extracted two types of metadata from the text: the general information on the publication and design-related parameters such as the materials used and device geometry corresponding to each curve. Finally, we wrapped the collected information into an explicit dataset record.

This paper presents a dataset of thermal emissivity of multi-material heterogeneous designs and the algorithm used for its creation. Regarding the dataset, this work describes the chosen data format and dataset organization. It also covers the technical validation of the collected records and provides a use case for the dataset. For the algorithm, the article presents a detailed description of the method used to collect every data entry. It addresses the aggregation of general information like DOI, publisher, authors, year, and title. Also, it covers the retrieval of materials, design, and method descriptions. Additionally, the paper reports the performance of a proposed tool for automated curve extraction.

## Methods

We established an algorithm that automates data extraction from figures and produced a comprehensive dataset of optical properties. Figure 1 provides an overview of the complete workflow; the various steps are described next in greater detail.

### Generation of initial corpus

We manually collected the corpus of 64 publications^21–84^ referring to emissivity by keeping track of relevant articles during our routine research for several years. We further used Google Scholar to search for articles by keywords and extracted more papers from the references. All selected articles contained graphical information of interest: emissivity-wavelength dependencies depicted as 2D curves on a blank background.

### Automated article information extraction from text

General information on publications (blue path and dataset component on Fig. 1) was extracted automatically using Mendeley^85^. We saved the corpus as a Mendeley archive, which allowed us to export it as a single BibTeX^86^ file containing the desired information. From the formatted BibTeX files, we used regular expressions^87^ to retrieve the DOI, title, authors, publisher, URL, and year of publication for each article. We note that dedicated software libraries for parsing BibTeX files such as pybtex^88^ (Python) are also available; however, we did not use those in this work.

### Manual design-related parameters extraction from text

**Fig. 2:**
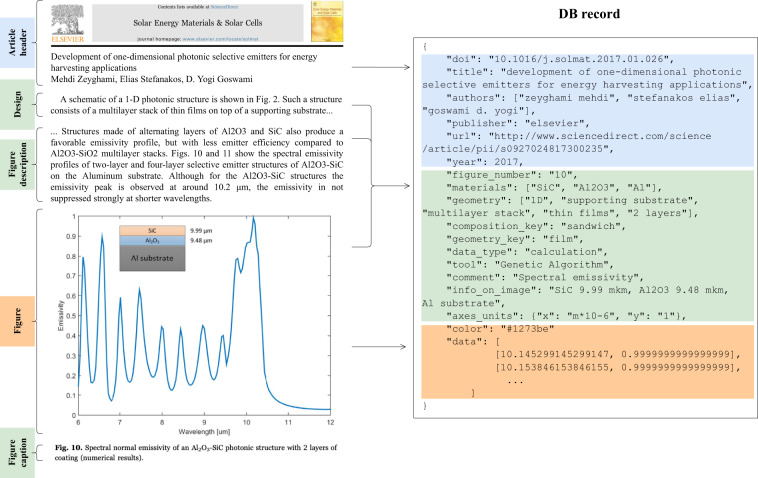
Information extraction from the source paper to the dataset record. Colors correspond to the category of extraction: blue - automated text analysis; green - manual text analysis; orange - automated figure analysis. Information is taken from different parts of an article; for example, materials are listed in the figure description and within the figure itself. This example uses the work of Zeyghami *et al*.^24^.

Design-related parameters (green path and dataset component on Fig. 1) are commonly reported in different sections of a publication, making it challenging to connect each curve record with its corresponding device geometry (sandwich, thin-film, grating), list of materials (W, Al, SiC), and method of data generation (calculation, experiment). Figure 2 demonstrates that design-related parameters of a given dataset record (data curve) were included in unrelated snippets of a sample paper. In the example from Fig. 2, the emissivity figure caption contains an incomplete list of materials and a brief geometry description elaborated in the figure-referring text. However, the full description of these two parameters is only given in the synthesis section of the paper, which already refers to other figures and does not mention the one with emissivity curves. We also note some complicated cases^49^ in our corpus when the authors reported the design solely graphically, leaving out the explanation in the text. Regarding the method of data generation, it could be reported in any location throughout the paper, and while it was usually possible to distinguish between experiment and theory from the context, many authors^23,34^ did not completely specify the used tool. For the issues listed above and others, our attempts to develop automatic tools for metadata extraction were insufficient to obtain the desired attributes (see SI.1), and this analysis was conducted through a manual approach.

We manually located text passages containing information about each curve and recorded this information. First, we recorded all distinct materials (chemical compositions) used in the device. We categorized records into two groups: “single material” if the structure was made out of a single material or “sandwich” if the structure was a multilayer design. We also parsed all keywords related to the device geometry. We found 100 distinct descriptors (thin film, aperiodic multilayers, 2D array, front coating). Using them, we classified geometry descriptions into seven types. These were: (i) film, (ii) 1D grating - a film with an array of slots of any shape on the surface with 1-dimensional periodicity (with or without coating), (iii) 2D grating - a film with an array of slots of any shape on the surface with 2-dimensional periodicity (with or without coating), (iv) 2D cylindrical cavities - a film with an array of cylindrical holes on the surface with 2-dimensional periodicity (empty or filled), (v) wire, (vi) bull’s eye - a film with a concentric equally spaced circular grooves on the surface (sometimes also with coating), and (vii) microspheres - random media composed of microscopic balls. Lastly, we parsed the method of data generation: experiment or computation and the characterization or modeling tool (Fourier transform infrared spectroscopy, finite-difference time-domain, etc.).

### Figure data extraction

The next step of the procedure was to detect emissivity records from graphs and parse them (orange regions on Fig. 2). We examined 64 papers for the graphical information of interest: emissivity-wavelength dependencies depicted as 2D curves on a blank background. We found 176 images with 550 thermal emissivity curves and manually converted them to PNG format. We manually split figures with multiple plots for the final one to contain a single plot panel and axis with units. The figures varied from 600 to 1400 pixels in width and 800 to 2000 pixels in height.

We followed a three-step algorithm for the automated extraction of structured data from figures (the orange box on Fig. 1). First, we identified the portion of the image with the axes and legend regions. Second, we looked at axes specifically and parsed the scale for the recalculation of pixel positions to units of measurement. Third, we removed the axes, legend, and gray objects (leaving just the curves themselves) and used a color decomposition algorithm to extract colored curve raw data. This procedure is fully automated if each curve is of a different color.

#### Axes and legend regions identification

We explored two approaches to detecting axis and legend regions: algorithmic and data-driven. Regarding the algorithmic methods, we tried Canny edge detection^89^ combined with the Probabilistic Hough line transform^90^ or polygon approximation^91^. We successfully found axes lines for 95% of figures in the dataset. However, these traditional methods expected fixed rules for each detected data type, making the algorithm brittle (see SI.2).

**Fig. 3:**
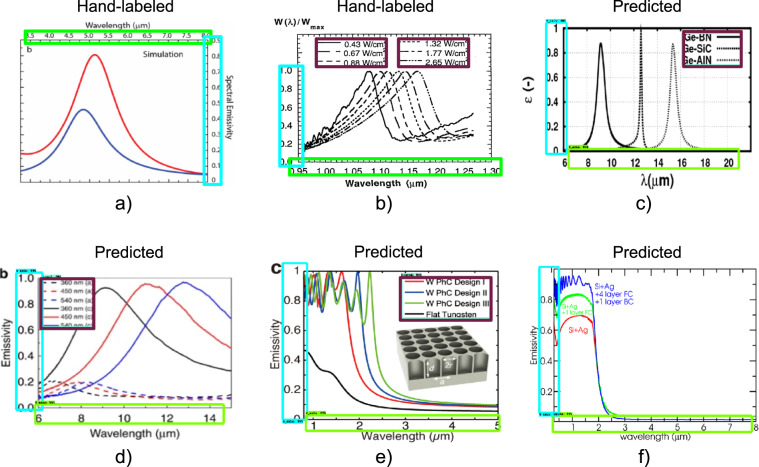
Examples of axes and legend labeling and trained CNN model performance. The x-axis is outlined in light green, the y-axis in cyan, and the legend in dark magenta. (**a,b**) Examples of hand-labeling using LabelImg^93^ software. Boxes depict the identified regions. Note that in b, the y-axis label includes just the portion with numbers and not the entire axis line for subsequent axis scale extraction; see text for details. (**c**–**f**) Examples of output of trained object detection model. Boxes demonstrate the detection results. For a: Copyright 1999–2021 John Wiley and Sons, Inc. All rights reserved. For b: reprinted from Timans, P. J. (1992). The experimental determination of the temperature dependence of the total emissivity of GaAs using a new temperature measurement technique. Journal of applied physics, 72(2), 660–670, with the permission of AIP Publishing. For c: Reprinted from Nefzaoui, E., Drevillon, J., and Joulain, K. (2012). Selective emitters design and optimization for thermophotovoltaic applications. Journal of Applied Physics, 111(8), 084316, with the permission of AIP Publishing.

For the data-driven approach, we used convolutional neural networks^92^ (CNNs). CNNs can detect multiple figure features using the same underlying framework but different data labels. We followed a standard procedure for supervised CNN learning with a pre-trained object detection model (first section inside the orange box in Fig. 1). We began by scaling all figures to the size of 800 × 600 pixels (only for the training of CNN; for future steps, we restored the original aspect ratio), splitting the set of 176 figures as 80/20 for training and testing and labeling all figures with LabelImg^93^ software. We labeled portions of images corresponding to the three classes: “X_axis”, “Y_axis”, and “Legend”. Axes regions required an axis line, ticks, and numbering. Legend regions included line samples and labels. Figure 3 shows examples of the labelling under a)^21^ and b)^80^. In the case of a), we located an X-axis region on top, a Y-axis region to the right, and no legend. Thanks to such images, the trained model can detect the axes objects with numbering on both sides of the axis line. In case b) of the Fig. 3, we identified axes regions containing numbering and two side-by-side legend regions aligned vertically for consistency. Overall, the trained model allows any number of legend objects, including zero.

After compiling the training data, we trained a machine learning model using Tensorflow 2^94^ (TF) object detection API^95^. We have a small dataset (for the comparison, the Microsoft COCO 2017^96^ object detection dataset has 121408 images), and using a pre-trained model from the Tensorflow Model Zoo is a powerful approach to handle this issue. Among the provided solutions for object detection, we selected faster_rcnn_inception_v2_coco model^97^ as it is lightweight with competitive accuracy. The model employs the Faster R-CNN^98^ attention mechanism and Inception Resnet^99^ deep convolutional network architecture, providing high-speed training. The model was pre-trained on Microsoft COCO 2017 images scaled to 600 × 1024 resolution. Training on our data with the default hyperparameters took 3.5 hours for 10,180 steps on 2.8 GHz Quad-Core Intel Core i7 processor running on a 2019 MacBook Pro.

We evaluated the model performance with standard metrics: precision, recall, and loss based on the intersection-over-union (IOU) method. In object detection tasks, IOU calculates a pixel-by-pixel difference of detected regions from human labels. Then, if we compare the detected region with the corresponding human label and calculate the area of the exact overlap, this value divided by the area of the detected object would be precision, and divided by the area of the labeled object would be recall. Loss sums up the localization loss (the undetected portion of the label area) and the classification loss (distinguishing between various classes). Following the training, Tensorboard calculated all these metrics on the test set. The model reached 0.75 average precision, 0.81 average recall, and 0.28 average loss (see the description of detection evaluation metrics used by COCO^100^ for more details regarding averaging). We note that perfect accuracy on these metrics is not required for the overall task of figure data extraction. Rather, we only need to detect enough of the axis information to be able to correctly perform axis scale parsing (see next step). Manual examination showed that all detected axis objects except one (99.4%) were acceptable in this regard.

Some example results of the model performance are depicted in Fig. 3c–f. In the case of c)^83^, the detected x-axis region missed the very left number; nevertheless, the captured information is enough to compute the axis scale, as will be described later. The model detected the y-axis region without issues. We note that the presence of other straight lines like the plot grid did not prevent the algorithm from identifying the axes correctly. Furthermore, the model correctly located the legend despite the unconventional location of the line samples to the right from the labels. In the case of d)^47^, all classes were correctly detected: x and y-axis regions contained axis lines, ticks, and all numbers, and the legend object included line samples and labels excepting the borderline. Case e)^101^ also had all objects of interest accurately located by the model. In f)^68^, the model slightly cropped the x-axis region, missing the last digit but capturing the majority of the numbering; the y-axis was fully detected.

#### Automated axis scale parsing

Following the identification of the axes and legend regions, the second step in the automatic data retrieval from the images (second section inside the orange box on Fig. 1) was obtaining the axis scale for recalculating pixel positions to units of measurement. We found that optical character recognition^102^ (OCR) methods were effective at axis numbering recognition with relatively few modifications or training needed. As the basis of our OCR strategy, we selected EasyOCR^103^. EasyOCR uses Pytorch^104^ for deep learning portions and Character-Region Awareness For Text^105^ for detection. For recognition, EasyOCR uses Convolutional Recurrent Neural Networks^106^ based on ResNet^107^, Long Short-Term Memory sequence labelling and Connectionist Temporal Classification^108^ decoding with the deep text recognition benchmark^109^. We adjusted EasyOCR’s model parameters, imposing a minimum height for the characters of 5 pixels and limiting character detection to numeric characters and special symbols such as a minus symbol or period. Also, we added white padding of 10 pixels from all sides of the images to reduce image edge impact and allow convolutions to operate better. Nevertheless, we note that some fonts were particularly unreadable for the model. EasyOCR returned a list of recognized numbers with the number value and pixel coordinates of the box with the number for every axis region. It properly handled 90% of our figures, producing sufficient information for scale calculation (detecting at least three numbers) for both axes.

To complete the axis scale recalculation, we cleaned EasyOCR output from the poorly detected values as follows. From the EasyOCR output, we filtered out entries with empty or non-numeric text and entries with the probability of recognition lower than 50%. Assuming that the numbers were centered correctly inside the detected boxes and on the tick lines (see SI.3), we approximated the location of ticks in the middle of the detected number boxes. Then, with automated rule-based approach, we applied a polynomial fitting for a set of tick pixel coordinates vs. recognized numbers, ensuring the fitting error subsided 5% for each instance and dropping outliers. We picked two middle points from the accepted set as they are typically more accurate than the edge ones and defined a linear equation for converting pixel coordinates to units of measurement.

#### Automated curve data extraction

**Fig. 4:**
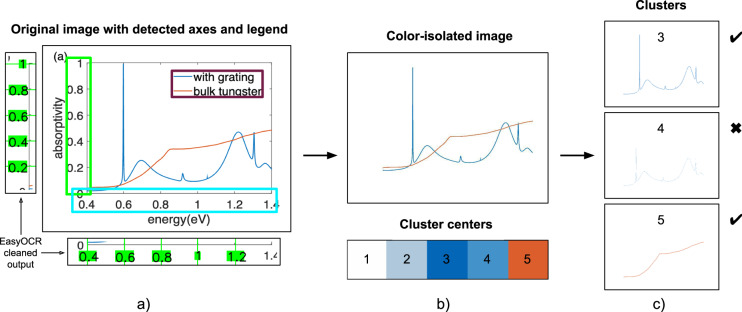
The pipeline for extracting axis scale and curves of different colors from figures. (**a**) Original image with detected x-axis (light green box), y-axis (cyan box), and legend (dark magenta box). Along the edges of the original image, we show the detected axes regions, the axes scale numbers as detected by EasyOCR^103^, and assigned green ticks. (**b**) On top is a color-isolated image that is the original image after removing the axes, legend, and black/gray objects. On the bottom is a color-isolated image palette with cluster centers determined by k-means clustering. (**c**) Data clusters of each color from the palette. Clusters 3 and 5 were accepted as they contain a single curve data. Cluster 4 was rejected as it contained only noise. After extracting the pixel coordinates of clusters 3 and 5, we matched them with EasyOCR cleaned output and converted to units of measurement.

For the final step of the automated figure data extraction, we parsed the colored emissivity curves using image color decomposition (third section inside the orange box on Fig. 1). We chose color decomposition because of the sophistication of this approach, although it has a room for improvement: it does not consider black curves, resulting in about half of the curves being excluded. Figure 4 outlines the framework of the color-based decomposition strategy. The first goal is to remove any extraneous plot elements apart from the data curves themselves. We removed black and gray objects such as axes, text, etc., by transforming images to the Hue Saturation Value (HSV)^110^ color scheme and whitening pixels with a Value or Saturation less than 50%. Also, we removed legends detected in the previous processing steps (dark magenta box on Fig. 4a)^23^ by coloring them white. This resulted in an image isolated to only colored pixels (top snippet on Fig. 4b). Second, we separated the obtained color-isolated images into clusters of different colors using k-means^111^ clustering (bottom snippet on Fig. 4b). We noticed that most of the existing solutions, such as the scikit-learn^112^ k-means package or Dominant Color Detection^113^, missed the colors corresponding to data curves (see SI.4). Therefore, we adjusted the k-means algorithm initialization (see SI.4). When examining the resulting clusters, some of the color clusters contained single curve records (clusters 3 and 5 on Fig. 4c), some had multiple curves information, and the rest had noisy data (cluster 4 on Fig. 4c). To filter some of the noisy data clusters, we removed clusters for which there were multiple y values for a single x value for more than 1/3 of the x data range. For example, this would exclude a color cluster in which both a solid blue curve and a dashed blue curve were present since the multiple curves would be detected as multiple y values for a single x value. Overall, we obtained clean raw data in pixels for 199 distinct curves.

Finally, we used the calculated axis scales (snippets on the sides of Fig. 4a) to convert pixel positions to units of measurement for the extracted curves. This results in 153 curve records in physical units (orange dataset component on Fig. 1), which determines the total dataset size. We note that we counted every pixel participating in a curve as a data point and did not perform any smoothing operations.

### Dataset generation

We applied the data extraction methods described above to the corpus to generate a dataset of thermal emissivity curves and associated metadata. In the case of multiple curves in a single image, we matched curves to metadata manually using a color name reference in the text. We normalized y-coordinates with values from 0 to 1 and standardized x-coordinates to micrometers. The total number of dataset records (one per curve) is 153.

### Unsupervised clustering of curve data records

To classify the 153 obtained curves into a manageable number of distinct groups, we applied an unsupervised clustering to the emissivity-wavelength curve data records stored in dataset. We set the wavelength range from 0 *μm* to 30 *μm* for all curve data records and reshaped the arrays to a size of 1000. Thus, each curve record became a 1-dimensional array of length 1000 with values from 0 to 1, where zero represented no emissivity measured. Next, we performed unsupervised learning with the DBSCAN algorithm implemented in the Scikit-learn library with parameters eps = 2.6, min_sample = 5 (see SI.5 for the hyperparameter search). The DBSCAN clustered half of the records (all the noise curves were put into a single class for the subsequent analysis, see SI.6) into seven classes of curves with close profiles in terms of Euclidian distance. More details regarding the results are in the Use case section.

## Data Records

The dataset of thermal emissivity records with metadata is represented as a set of JSON files and may be found at Figshare^114^. Table 1 provides an overview of the data record schema. The first set of keys refers to article-related attributes: DOI, title, authors, publisher, URL, and year of publication, and also the figure number given in the paper. The remaining attributes are curve-related: list of materials, keywords describing geometry, measurement or calculation method, legend information, axes units, color, score (see Technical Validation for details), and curve raw data. One JSON file corresponds to information retrieved from a single curve. See SI.7 for possible values for various keys.

**Table 1:** Format of data records in dataset.

	KEY	DESCRIPTION	DATA TYPE
article-related	doi	DOI of the source paper	String
	title	Title of the source paper	String
	authors	Authors of the source paper	List of strings
	publisher	Name of the publishing group	String
	url	Link to the paper	String
	year	Year of paper publication	Integer
	figure_number	Name of figure appearing in the source paper	String
curve-related	materials	All materials used in the sample	List of strings
	geometry	Keywords from the geometry description in paper	List of strings
	composition_key	Assigned keyword: sandwich or single material	String
	geometry_key	Assigned keyword: one of the 7 geometry classes	String
	data_type	Calculation or experiment	String
	tool	Equipment, software or theoretical approach	List of strings
	info_on_image	Additional information appearing on image	String
	axes_units	Units of X and Y axes	Dictionary
	color	HEX color code of the curve	String
	data	Raw curve data in a form of list of [X,Y] coordinates	List of [float, float]
	score	Score of curve from Technical Validation	Float

## Technical Validation

We evaluated the efficiency (recall) of the automated figure data extraction pipeline by the portion of the curves extracted from the total number of curves in the data set. Algorithm obtained 153 single curve records. The studied images contained 550 curves, of which half curves were colored curves. The total efficiency over all curves was thus 27%, whereas the total efficiency over colored curves was 55%.

We also studied the quality of the extracted curve data records. To define a quality score, we considered two types of failures in the curve record: gaps in the data and multiple (conflicting) points. Data gaps in a curve frequently occur due to overlapping objects of different colors on the extracted curve and result in x coordinates without corresponding y values. Multiple conflicting points typically appear when the original image had text comments or symbols of the same color as the curve. Such data points contain multiple y values (taking into account the line thickness) for a single x coordinate. We note briefly that attempts to clean text with OCR algorithms often produced gaps; for example, OCR assigned the oscillating portions of data curves with the letter M with very high confidence scores (see SI.8).

Using the failure types defined above, we assigned every x data point of the record as “correct”, “gap”, or “multiple”. We note that small gaps (running for less than 2% of the curve length) were assigned as “dash” to avoid low scores for the dashed style curves. We evaluated the quality of each curve data record, calculating the portion of X data range with correctly extracted points using the Eq. 1:

1 $$Score=1-\frac{{N}_{GAP}+{N}_{MULTIPLE}}{{N}_{GAP}+{N}_{DASH}+{N}_{MULTIPLE}+{N}_{CORRECT}}$$

**Fig. 5:**
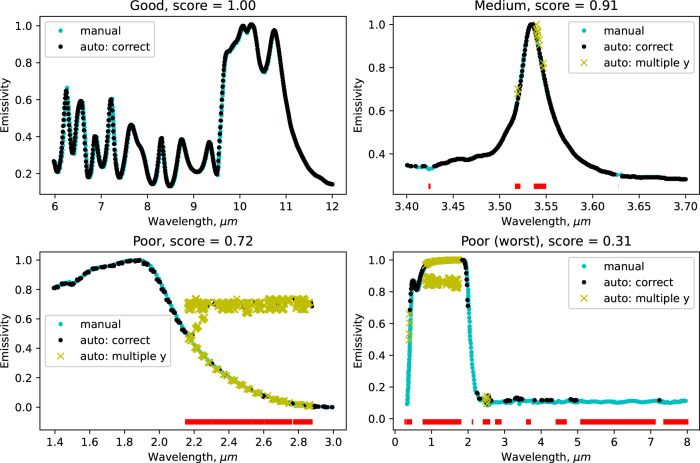
Several example curves and comparison between automated (black dots for correct points and yellow crosses for multiple y points) and manual (cyan) extraction. The red line on the bottom depicts the unconfident area, demonstrating the portion of the curve where an extraction has failed (gaps, multiple y values). The scores were calculated with Eq. 1. Top left: good extraction, score 1.00, the algorithm correctly captured the entire curve region. Top right: medium quality of extraction, score 0.91, the record has a few gaps and multiple y points. Bottom left: poor extraction, score 0.72, the original curve has dashed style; many multiple y points created by text comment. Bottom right: poor extraction, the lowest score of 0.31; many gaps caused by overlapping, multiple y points due to text comment.

We also performed hand labeling (using the WebPlotDigitizer^15^ software) for curves at different scores and compared the extracted and actual records. The error of the manual extraction was around 2 pixels representing the click accuracy. Figure 5 plots both manual and automated extractions for four cases. The first example is a curve with a score of 1. Automatically extracted points accurately matched manual extraction. The second example is a curve with a score of 0.91. It contains some gaps and a few instances of multiple y points, but most of the curve is extracted correctly. We assigned this record to be of medium quality. The third is a curve with a score of 0.72. Although we correctly handled the dashed style of the curve, this record originally contained a large text comment producing a significant amount of failure data points. This record is considered a poor extraction. Finally, the curve record with the lowest score of 0.31 contained very large gaps and a massive portion of multiple points among the extracted data. Fortunately, the dataset contained only a few records in such a condition.

**Fig. 6:**
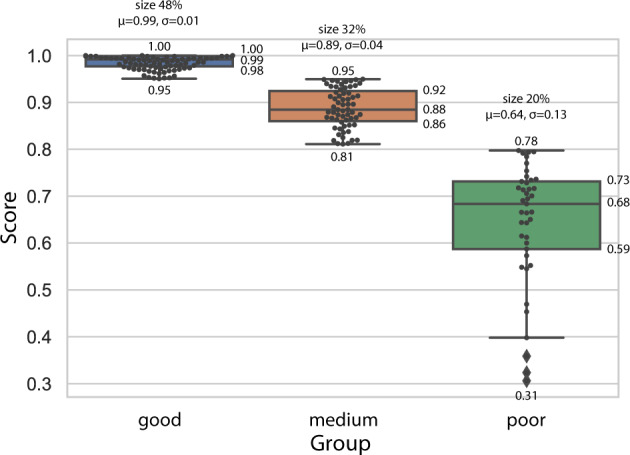
Statistical analysis of extracted curve data records grouped as good, medium, and poor. Each point represents one curve, and scores show the quality of the extraction. Scores were calculated with the Eq. 1. A mean *μ* and standard deviation *σ* values are given on top, along with the relative size of each group.

We grouped the data curve records by the calculated score: good curves with scores exceeding 0.95, medium curves from 0.8 to 0.95, and poor curves for scores below 0.8. Figure 6 depicts the behavior of each group. Approximately half of the records were good, one-third were medium, and one-fifth of the curve records were bad. There were 40 records with scores above 0.99; the worst entry had a score of 0.31. All in all, the proposed automated curve data extraction algorithm produced 122 (80%) good and medium-quality records.

### Use case

**Fig. 7:**
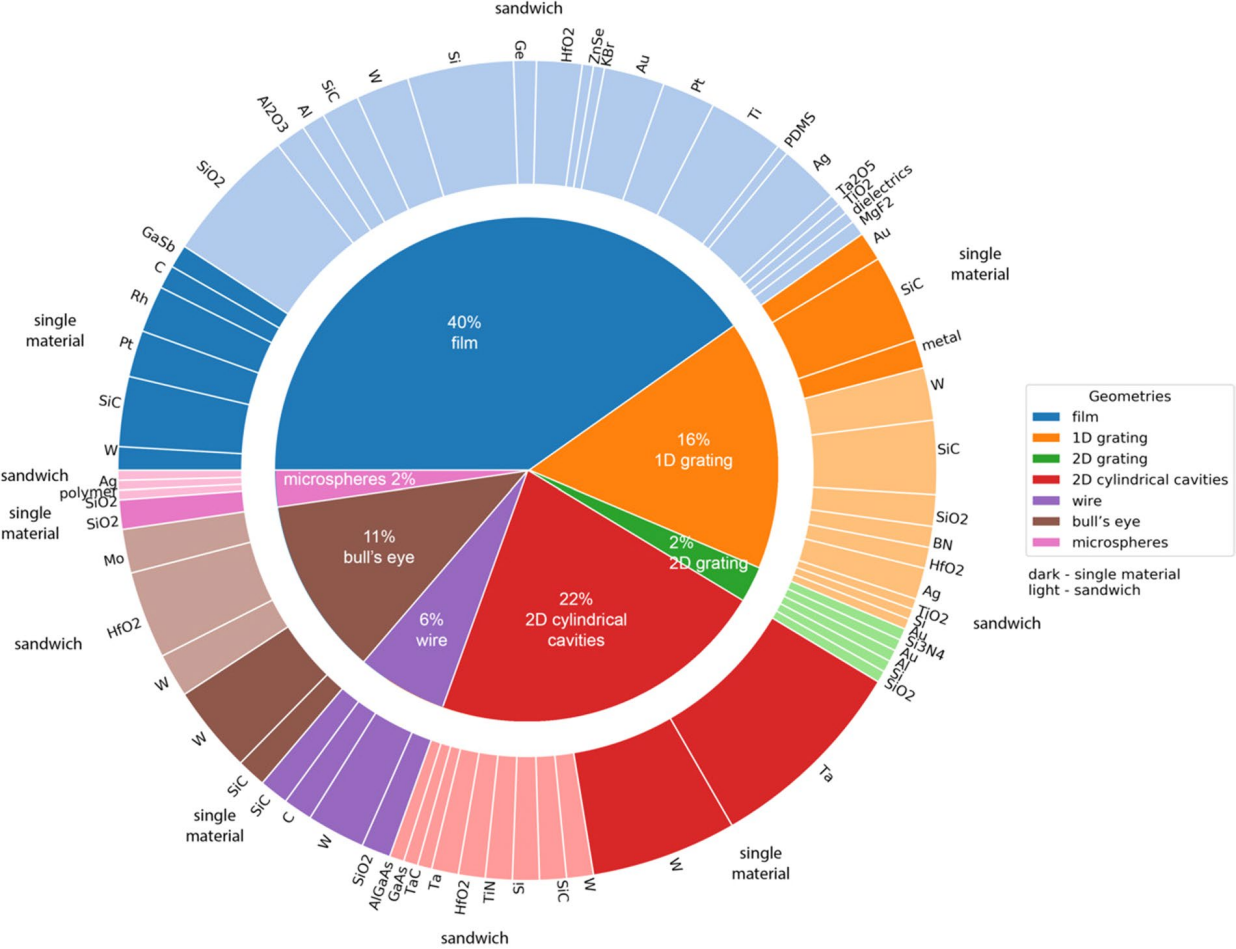
The distribution of design-related parameters (geometry, materials) in the dataset. The innermost circle corresponds to geometry. The outer ring depicts the used materials with colors reflecting the composition: the color is dark for single material devices and light for sandwich structures. There are 32 distinct materials. In total, there are 60% sandwich and 40% single material structures. The most used material overall is tungsten, which has desirable properties for optical devices.

Next, we examined the data set to understand the overall distribution of the data. This analysis is plotted in Fig. 7. In our dataset^114^, the most often used design was a sandwich film. Because this structure is a 1D multilayer stack of thin films, it is easy to model and fabricate. Nevertheless, tuning the composition of the layers, the number of layers, and the thickness of each layer to obtain the desired radiative properties remains a challenge^24^. Another common design in our dataset was a single material slab with a 2D array of cylindrical cavities on the surface. 2D all-metallic emitters are better suitable for high-temperature applications than multilayer structures due to higher chemical and mechanical stability. Also, a 2D grating provides a higher surface-to-volume ratio increasing the emissivity. Grating period, depth, and shape are commonly used to tune the emittance spectrum^48,115^. Analyzing the materials, we found tungsten to be the most popular choice. This is a reasonable observation. Tungsten has the highest melting point among all pure metals and favorable optical properties for selective emitters, such as high emission up to a cutoff wavelength and very little beyond. That makes tungsten a desirable choice for optical samples operating under high temperatures^29^. However, when considering the complete optical device design, the most common configuration was tantalum film with a 2D array of cylindrical cavities on the surface. Tantalum has similar optical properties to tungsten with a high melting point, low vapor pressure, and long-wavelength emissivity (above 2 *μm*). Also, it is weldable and machinable^48^. Other common configurations include using silica as a sandwich layer^34^ and the use of tungsten films with a 2D array of cylindrical cavities^60^. We note, however, that these attributes may change depending on the particular data set of papers used as the source.

**Fig. 8:**
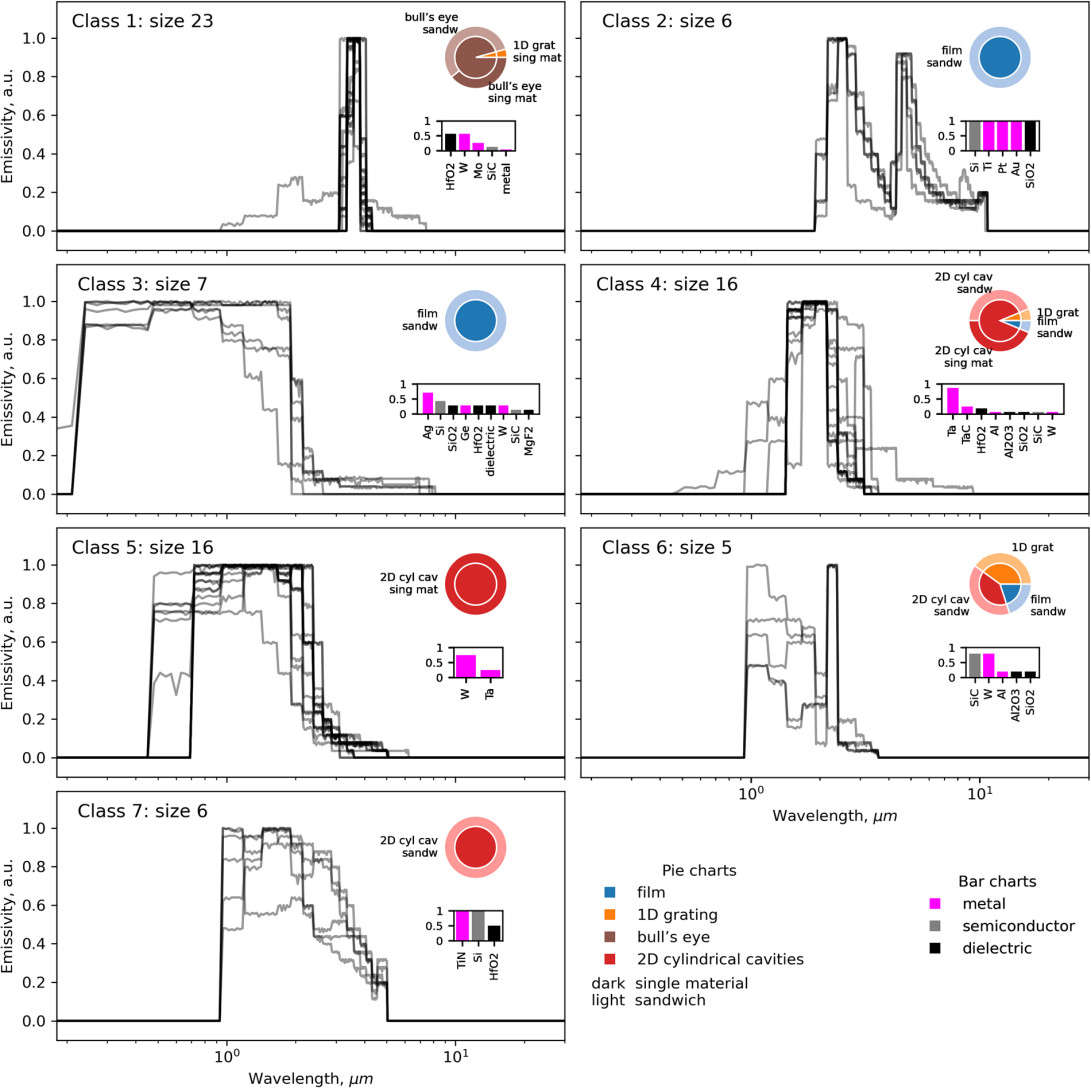
Curves classes with similar emissivity behavior and distribution of corresponding metadata. Curves were clustered with unsupervised learning using the DBSCAN algorithm. Curves are plotted with partial transparency such that dark areas indicate overlap of curves. The x-axis is in logarithm scale for better visualization. Pie charts in the insets show the distribution of geometry and composition per class. Bar charts in the insets depict distinct material frequencies normalized per class size (i.e., if the bin height is 1, the material is present in every record in the class).

Next, we aimed to find trends in emissivity profiles and correlate them with device attributes. We followed an unsupervised clustering strategy to identify groups of emissivity curves with similar behavior (see Methods section) and analyzed metadata within each group. Figure 8 shows that the agreement inside each group is generally good: curves are plotted with partial transparency, and the darker regions correspond to curve overlap. Each class depicts a unique emissivity behavior. We note that class 6 had a variety of samples and therefore was nonuniform. All classes except 6 had a single dominant design and composition with few outliers (pie insets in Fig. 8) as well as dominant materials (bar insets in Fig. 8). We observed that class 1 had the sharpest peak compared to others. Most class members had a bull’s eye structure which is indeed designed for thermal beaming. A series of equally spaced circular concentric grooves produces an emission spectrum in the normal direction with a single peak at a wavelength nearly identical to the period^62^. Class 2 forms a bi-modal emissivity profile. Sandwich films, in this case, were designed as Fabry-Perot cavity resonators^116^. They contained *Si*, *Ti*, and *Pt* layers covered with opaque (thick) *Au* layer, *SiO*_2_ cavity layer, ultra-thin top *Au* layer, and *SiO*_2_ protection layer. Fabry-Perot cavity resonators produce two emission peaks at locations determined by the optical properties of the cavity, opaque and top layer materials (*SiO*_2_, *Au*), and the cavity’s thickness. Peaks amplitudes are sensitive to the thickness of the top layer^54^.

As Fig. 8 demonstrates, geometry seems to be the major factor defining curve behavior. Designs without any grating on the surface ended up in classes 2 and 3. Records with a bull’s eye geometries fell in class 1. If there was a 2D periodic grating on the surface, we obtained classes 4, 5, and 7. The selection of materials further defined the curve behavior. Usage of *Au* and presence of *SiO*_2_ cavity determined class 2, while class 3 members were multilayer stacks of cermet layers with a *Ag* reflective back^29^. Similarly, *Ta* was characteristic for class 4, *W* for class 5, and *TiN* for class 7. We trained a decision tree (see SI.9) that further demonstrated primary splitting of behavior on geometrical attributes and secondary splitting on a choice of materials.

### Other work

A dataset of emissivity curves was previously reported by Frolec *et al*. in 2018^117^. It contains 58 records of thermal emissivities experimentally measured by authors starting at cryogenic temperatures and slightly exceeding room temperature. The curves were obtained from 45 different samples covering a range of pure metals, alloys, foils, coated metals, and ceramic plates. The dataset does not contain spectral data but provides information on the bulk material, coating layer material, treatment techniques, and the temperature dependence of the hemispherical emissivity or absorptivity. In contrast to the current work, the information was not compiled from the literature but rather was measured by the authors. Thus, this dataset represents more consistent techniques for data generation but is more limited in scope and delivers less information.

Another work from Kobayashi *et al*. presents normal spectral emissivity dataset measured at high temperatures^118^ reaching 1500 K. This work also includes the results of measurements performed by the authors in the same laboratory and the dataset does not provide spectral data. All the investigated materials were metal surfaces with different degrees of oxidation, and the surface roughness is stored as one of the parameters in the records. Seven different metals were studied, and only a single design was represented. Thus, while the consistency of the method is higher than the one we report, the scale and diversity of data are more limited.

Our dataset contains spectral data and corresponding materials, method and design parameters. In our dataset, the temperatures usually lie between room temperature and 2500 K, although we have not rigorously parsed all the temperature values for all curves (information regarding the temperature is sometimes contained within the “info_on_image” key in the JSON records). Emissivity - wavelength data relations are both experimental and theoretical obtained with different equipment. We store many designs of different complexity and details regarding the sample geometry. There are 32 distinct materials. Thus, our dataset does not have any records fully duplicating those mentioned above or any other work, advancing previously published emissivity databases.

In this work, we manually collected 64 papers with 176 figures reporting emissivity and automatically extracted 153 curve records with manual extraction of corresponding metadata. However, there exist more publications reporting emissivity. We analyzed the collected metadata in our dataset with the CountVectorizer package featured in Scikit-learn library without tokenization^119^ and found that only 45% of captions of relevant figures mention emissivity, while 88% of captions mention any of emissivity, emission or emittance (9% mention both emissivity and emission, 17% mention only emission, 26% mention only emittance). Thus, among relevant entries, 88% satisfy the criteria of three words; and half of interesting figures may be missed if we excluded terms emission or emittance from the search. Then, we checked manually that among 100 random figures satisfying the criteria of three words (mentioning emissivity, emission or emittance in the figure caption), 70% indeed presented thermal emissivity curves. To determine how many more figures might be possible to collect, we implemented nine electronic paper scrapers (see SI.10) that checked figure captions amongst 4.9 million papers for the above keywords. As an aside, we note that there are packages like EXSCLAIM!^120^ to help automate this process, but it does not support the journal publishers targeted in our work. Our results indicate that there exist 361,000 figures (178,000 papers) mentioning emissivity, emission, or emitter in the caption and potentially 70% of them would have the data we want. While we envision it may be possible to use an automatic curve data extraction algorithm to obtain a reasonable fraction of these curves, extracting the design-related parameters from text (i.e., the geometries, materials, and methods that describe each curve) remains a challenge and the biggest bottleneck for expanding the optical properties databases.

The automated generation of databases incorporating textual and spectral data has several remaining challenges. First is corpus composition, which includes an automated selection of papers relevant to specific tasks; it can be accommodated with existing text-mining tools^121^. The second is metadata extraction, and advanced text-mining algorithms fine-tuned for specific applications are promising for this task^122^. The next challenge comes from curve extraction with a color decomposition strategy; as described previously, OCR routines fail to distinguish between curve and text comments of the same color and, therefore, cannot be used to exclude text data. Furthermore, black curves cannot be isolated since the curve detection routine removes all grayscale pixels (e.g., to remove axes) prior to curve isolation. Also, dashed and solid curves of the same color cannot be differentiated. More advanced methods, such as image segmentation, may be able to overcome some of these limitations.^94^. The other is extracting the additional information from figures, such as linking legend labels with curves, that has been partially addressed in other methods^20^.

## Usage Notes

The set of JSON files is available at Figshare^114^. Each file can be opened with any software for text editing or by common programming languages. The python script for re-plotting the data from any of the JSON records is available at https://github.com/ViktoriiaBaib/curvedataextraction and called “replot_DBrecord.py”. The repository also contains some scripts for querying the dataset for the presented analysis and beyond.

## Supplementary information

Optical emissivity dataset of multi-material heterogeneous designs generated with automated figure extraction Supplementary Information

## Data Availability

The source code (implemented in Python) for performing all the described figure analysis steps and generating the data entries is available at https://github.com/ViktoriiaBaib/curvedataextraction. The axis and legend detection step uses the TensorFlow2 Object Detection API and provides a fine-tuned CNN model. File “object_detection_axes_legend.py” performs object detection of legend, x-axis, and y-axis objects and generates PNG and JSON records for these objects. File “color_decomposition.py” performs clustering by color and produces PNG of color-isolated image, palette, as well as PNG and JSON records of separate color clusters in pixel coordinates. It uses methods from “posterization.py”. File “final-record.py” performs axes scale parsing and applies it to all the clusters, producing cluster records in units of measurement. It utilizes methods from “final_record_func.py”.
