# Supplementary material for: Optical emissivity dataset of multi-material heterogeneous designs generated with automated figure extraction
Source: Sci Data. 2022 Sep 29;9:589. doi: 10.1038/s41597-022-01699-3 (PMC9522672; doi:10.1038/s41597-022-01699-3)
Supplement: Supplementary file 1 — Optical emissivity dataset of multi-material heterogeneous designs generated with automated figure extraction Supplementary Information [file 41597_2022_1699_MOESM1_ESM.pdf]

Optical emissivity dataset of multi-material heterogeneous designs generated with automated figure extraction Supplementary information

Viktoriia Baibakova[1], Mahmoud Elzouka[1], Sean Lubner[1], Ravi Prasher[1], and Anubhav Jain[1*]
[1]Lawrence Berkeley National Laboratory, 1 Cyclotron Rd, Berkeley, CA 94720, USA

*Corresponding author: Anubhav Jain; ajain@lbl.gov

Contents

1 Automated retrieval of design-related parameters from text 1

2 Algorithmic approach to axes regions identification 1

3 Ticks location during the automated axes scale parsing 2

4 Image color decomposition: other methods and our approach 2

5 Search for the best parameters for unsupervised clustering of curve profiles with DBSCAN 3

6 Curves left out as noise by DBSCAN 4

7 Possible values for various keys in data records 4

8 Rinsing text off with OCR algorithms 5

9 Decision tree for metadata analysis 6

10 Nine electronic paper scrapers 6

1 Automated retrieval of design-related parameters from text

Automatic tools were insufficient to obtain the desired design-related attributes from the text. Figure captions and in-text figure-referring descriptions were easy to locate but lacked much data. To examine how much information we could automatically retrieve from them, we processed the collected captions and descriptions with the Lawrence Berkeley National Lab Natural Language Processing (LBNLP) package [1]. LBNLP includes standard text mining tools for materials science and chemistry using the pre-trained inorganic materials model [2] for Named Entity Recognition [3] via Long Short Term Memory neural network [4]. The algorithm identifies materials, properties, applications, phases, structure descriptors, synthesis, and characterization methods assigning corresponding tags to the words. However, LBNLP is not specific to the optics domain. Applied to our data, LBNLP highlighted descriptors quite broadly, complicating future studies. Materials and geometries found by LBNLP significantly differed from the manual check for each record. Automatic extraction mistakenly defined a silicon carbide film as the most used design. However, this approach managed to highlight tungsten and tantalum slabs with a 2D array of cylindrical cavities on the surface.

2 Algorithmic approach to axes regions identification

We used computer vision algorithms implemented in the OpenCV [5] package to localize axes lines on scientific plots. We tested two approaches. First was the Canny edge detection [6] combined with polygon

approximation [5]. It detected axes box, drawing the rectangular on top of the four axes lines (all of which had to be present on the plot for this approach to work). It located the axes box on 63% of figures, and all of them were correct (sometimes slightly displaced). The second approach used Canny edge detection combined with the Probabilistic Hough line transform [7]. It detected each axes line separately, aiming to locate x-axis and y-axis lines. This approach found lines on 92% of figures from our set, but it made mistakes such as confusing grid lines with axes lines. Then, we applied both approaches sequentially and correctly found axes lines for 95% of figures in the dataset. The localization of axis lines required a large amount of custom code, leaving out the numbering and ticks detection. Also, this approach is unreliable as different images will likely result in new issues to handle. All in all, the traditional methods performed in a manner that suggested they would not be robust to future changes.

3 Ticks location during the automated axes scale parsing

During the development of the automated axis scale pipeline, we used the EasyOCR [8] package for axes numbering detection and recognition and assumed that ticks were located at the center of the detected number box. This assumption is valid if two conditions are satisfied: (i) EasyOCR must correctly localize the number region with a tight box; (ii) ticks must be centered to numbers. A manual check proved that in most cases, both conditions were true. Figure 1 shows some examples of axes scale parsing with the original axes region and the result of automated axes detection next to each other. Assumed (green) ticks closely match with the original ticks.

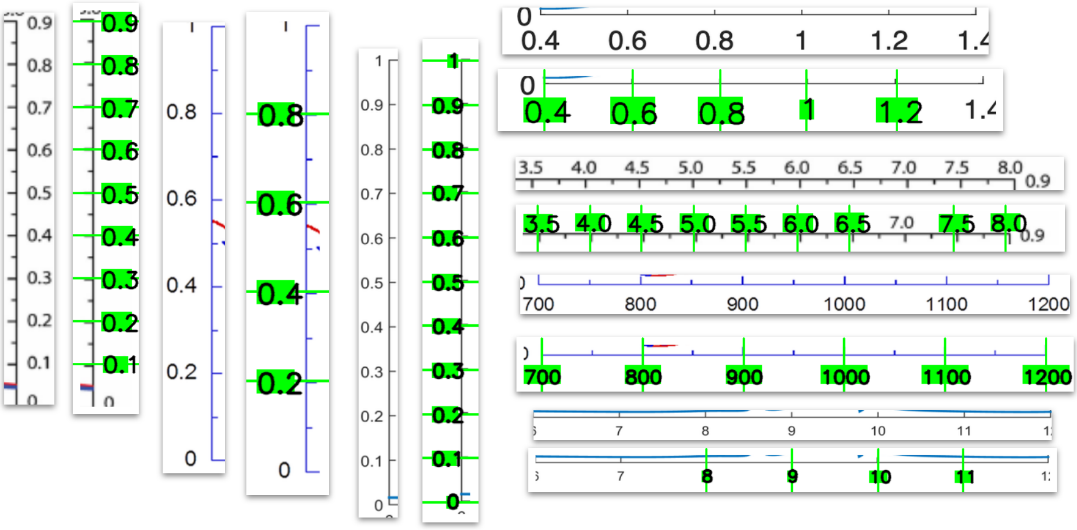

Figure 1: Examples of axes scale parsing. Only accepted numbers are shown. Green box is a number region detected by EasyOCR, black number on top of it is a number recognized by EasyOCR, green line depicts the center of the green box and corresponds to the assumed tick location for the recognized number.

4 Image color decomposition: other methods and our approach

We noticed that most of the existing solutions, such as Color Thief [9] tool, Scikit-learn [10] k-means package, Dominant Color Detection [11], missed the colors. Figure 2 shows the incomplete palettes produced by each of the listed methods. Also, the palette changed every time we applied the mentioned method. We assumed that the issue was caused by the random initialization of the color centers. We have mostly white images, and it is statistically difficult to get complete diversity of colors in one random set.

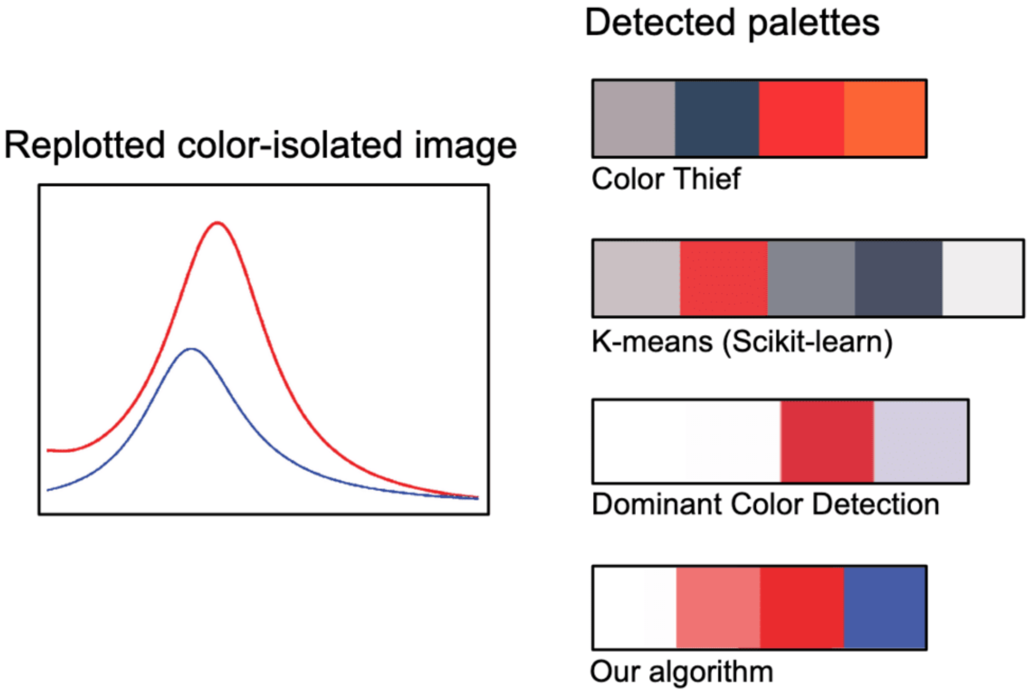

Figure 2: Palettes (color centers) produced by different methods. The methods were applied to a color-isolated image of the replotted figure with 2 curves of red and blue colors. First three palettes miss the blue color while the palette produced by our algorithm has it.

We have adjusted the k-means algorithm initialization, forcing it to start with the eight color cluster centers representing a combination of RGB and CMYK modes: white, red, green, blue, cyan, magenta, yellow, and black. Then, we iteratively updated the palette, checking the distance between every pixel and color cluster centers (L2 norm in RGB space). Also, we allowed dropping the empty clusters. This modification resulted in a correct steady set of color centers for each image. Figure 2 shows the palette obtained with our algorithm.

5 Search for the best parameters for unsupervised clustering of curve profiles with DBSCAN

DBSCAN method [10] has several parameters to adjust. First, eps - the maximum distance between two objects for being considered as neighbors. Second, min_samples - the neighborhood's minimum number of members (or total weight). The third is the metric for distance matrix calculation. The metric did not significantly influence our result, so we set it to be Euclidian and focused on searching for the best values of eps and min_samples. We analyzed how the number of clusters and the number of unclustered curves (noise) depend on these parameters. When eps was less than one, only a small portion of samples was clustered, and the noise cluster was large. An increase in eps increased the number of clusters, reducing the noise. However, a further increase in eps reduced the number of clusters as the extracted groups started to concatenate. There was no noise when eps was equal to five, and all entries were put in a single cluster. All in all, we determined that the best values for parameters were eps = 2.6 and min_samples = 5, which produced 7 clusters leaving half of the curves as noise. Notably, min_samples influenced the number of clusters much stronger than the noise volume. Supposedly, a change in cluster size did not involve new samples to be clustered, simply refining the existing clustering. We map the clusters with the package UMAP [10] in Figure 3. One dot corresponds to a curve; colors correspond to the cluster labels. Although the axes units are noninterpretable, all clusters are well defined in this mapping.

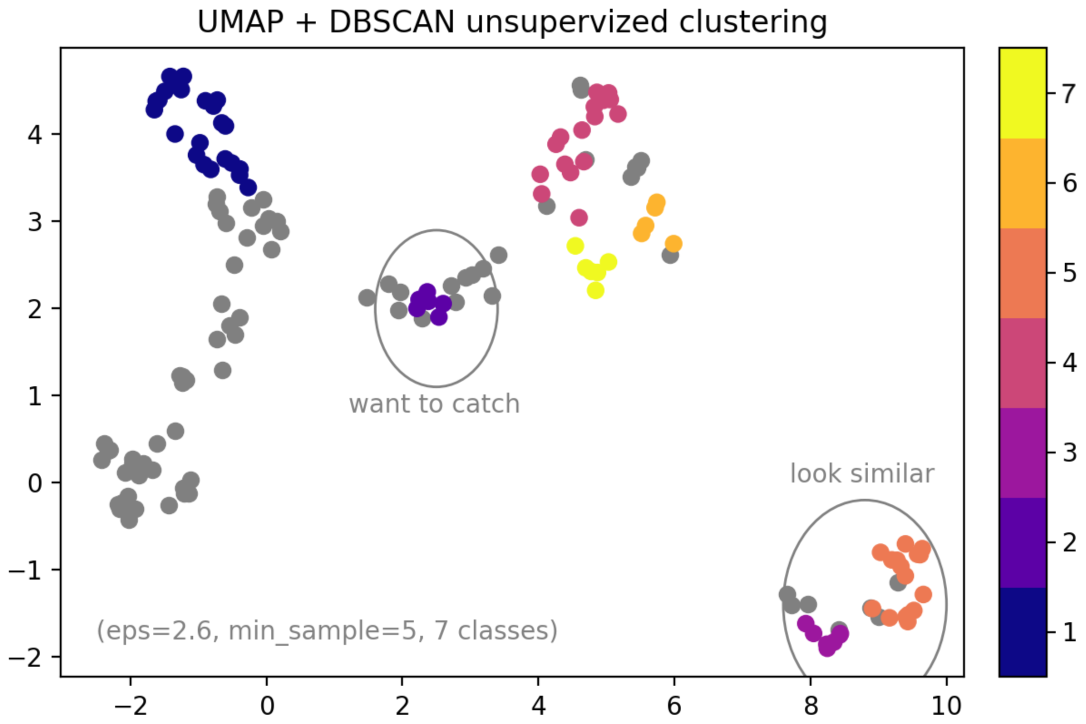

Figure 3: Mapping of the labels assigned by the DBSCAN algorithm. There are seven different classes colored, and the gray points relate to noise.

6 Curves left out as noise by DBSCAN

Unsupervised clustering with the DBSCAN algorithm found groups of similar behavior among half of the records, labeling the other half as noise. We put the noise curves into a single class. Figure 4 demonstrates that the noise class curves have a variety of profiles.

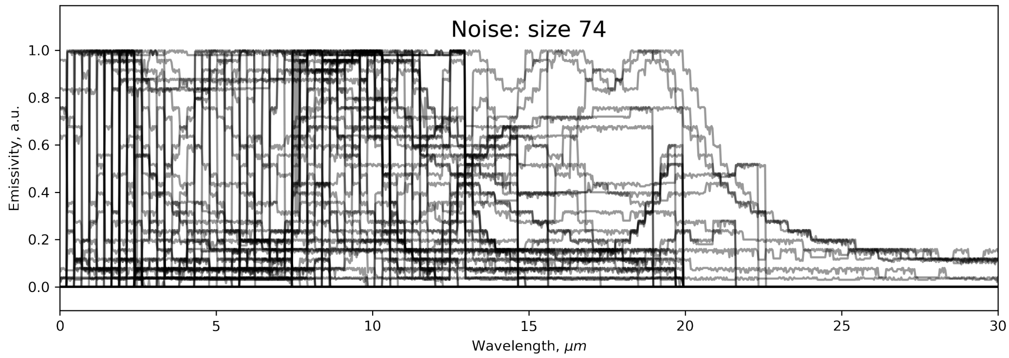

Figure 4: All unclustered curves labeled as noise by DBSCAN. Curves are plotted with partial transparency.

7 Possible values for various keys in data records

The dataset of thermal emissivity records with metadata is represented as JSON files with various keys. Table 1 provides possible values for the keys in data records. The content is not limited for some keys, and the value can list any number of descriptors. Other keys can have only one value out of a fixed set. Regarding the unlimited values, key "geometry" stores all keywords used in the source paper for the characterization of the geometry, which we considered to be descriptive. Also, under key "materials", we listed all materials used in the device. In contrast, we put a single value from the chosen set for

”composition_key” and ”geometry_key”. Key ”data_type” can have one of two values, but the key ”tool” lists all mentioned methods. Key ”comment” is for any important notes; key ”info_on_image” stores information from a figure given as a text comment or in an inset. Key ”color” provides a HEX color code of the cluster center found with the automated curve data extraction algorithm. Key ”score” contains value of a quality score estimated during technical validation with values from 0 to 1.

KEY	POSSIBLE VALUES
materials	*any set from:* W, SiO2, SiC, Si, HfO2, Ag, Au, Ti, Ta, Pt, Al, Al2O3, Mo, Ge, MgF2, TiN, Rh, TiO2, BN, C, GaSb, AlGaAs, GaAs, metal, dielectric, Ta2O5, TaC, PDMS, KBr, ZnSe, polymer, Si3N4
geometry	*any set from:* 2D array, sandwich, multilayer, wafer, circular concentric grooves, bull’s eye structure, metallic-dielectric, coating, cylindrical microcavities, cylindrical cavities, aperiodic multilayers, adhesive layer, barrier layer, thick film, ultra-thin film, cavity, PC slab, vaccum gap, flat surface, 1D grating, multilayer stack, a cavity hole, thick disc, vacuum gap size, period, grating depths, 1D periodicity, multilayer aperiodic, infinitely long, cylinder, thin films, 1D, square lattice, n-type doped, 6H, thin film, 4 layers, 9 layers, front coating, back coating, thin barrier layer, mirror, multilayers, roughness, PhC, flat disk, ZGS, alloy, microspheres, 5 layers, 6-8 layers, dielectric, semiconductor, back reflector, metal-dielectric composites, cermet layers, dielectric AR coating, 2 layers, cylindrical holes, porous, random media, p-n junction, gutter, metal slab, corrugated, grating, 1D array, 90 layers, square piramids, thick layer, surface degradation, nanowire, 7 layers, filled microcavities, ceramic plugs, polymer, 3 layers, semi-infinite layer, binary layer of dielectrics, unit cell, metal coating, 1D PC, AHM, tilted optical axis, hyperbolic metamaterial, mesh of orthogonal slits, 1D grating; 2D grating, taper, periodic slits, hybrid metamaterial, glass-polymer, AR coating, flat, 1-4 layers, 10 layers, grating slab, 60 $\times$ 60 unit cells, square, lithographically patterned into a Babinet metamaterial, sub-wavelength thick layers
composition_key	*one of:* single material, sandwich
geometry_key	*one of:* film, 1D grating. 2D grating, 2D cylindrical cavities, wire, bull’s eye, microspheres
data_type	*one of:* calculation, experiment
tool	*any set from:* FDTD solver, CST 2014 microwave studio, Finite - difference time - domain (FDTD) simulation, Transfer matrix method (TMM), Bayesian optimization with COMBO, UV-vis, RCWA, UV-Vis-NIR, Utraviolet/visible/near-infrared spectrometer, finite difference time-domain (FDTD) simulation, spectrophotometer, Jasco V-670 with an un-polarized light source, genetic algorithm, GA, spectrophotometer with an integrating sphere (Vertex 70, Bruker), CAMFR, monochromator, FDTD simulations, Genetic Algorithm, analytical, numerical simulation, mispherical-directional sphere reflectometer, MEEP, theory, FTIR spectrometer, high-temperature emissiometer, FDTD, FTIR

Table 1: Possible values for metadata keys in data records.

8 Rinsing text off with OCR algorithms

Some figures have the text comment of the same color as the curve, and in these cases, one color channel contains both curve and comment information. The comments are of very different content: sometimes it is a word from the standard English language, sometimes it is a sequence of Greek letters or an equation with numbers and mathematical operators. In an attempt to remove the text, we used EasyOCR [8] package allowing any English and Latin letter as well as numbers and symbols. EasyOCR detected text comments but often returned meaningless messages due to the high diversity of allowed symbols. Also, for the curves of complicated behavior, EasyOCR mistakenly detected portions of the curve as text. Figure 5 shows an example of detection with incorrectly recognized text comments and letters ”I” and ”M” assigned to oscillating parts of curve.

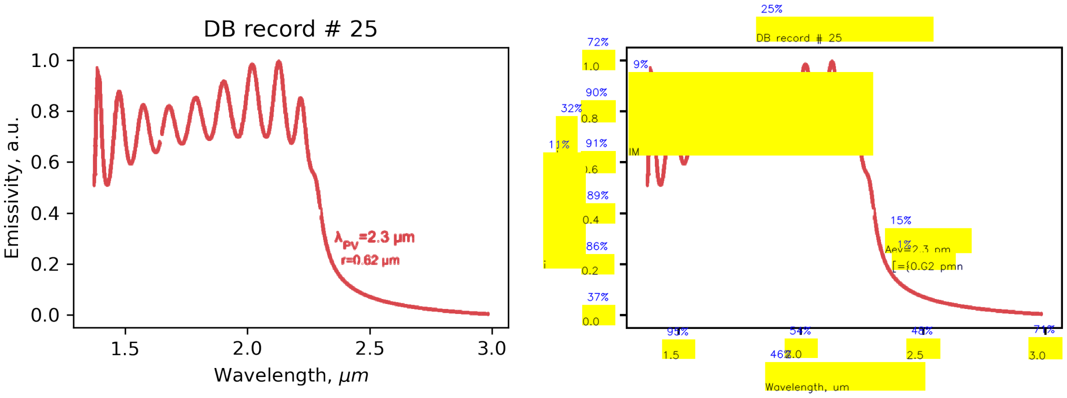

(a) replotted curve record

(b) EasyOCR recognition result

Figure 5: Example of EasyOCR output when applied to the curve with complicated behavior. a) Plotted DB record containing curve with complicated behavior and text comment of the same color. b) Plotted DB record with EasyOCR output; yellow regions show areas where EasyOCR detected any text, black text on top of the yellow regions is a result of EasyOCR recognition, and blue text above the yellow regions is a certainty of recognition.

9 Decision tree for metadata analysis

To better understand the role of metadata for the curve classes, we trained the decision tree with Scikit-learn library [10]. For the geometry, we used a numerical encoding: 0 - film, 1 - 1D grating, 2 - bull's eye, 3 - 2D grating. The other geometry types were present only in the noise class. We applied a binary encoding for the composition: 0 - single material, 1 - sandwich. For the material list, we used one-hot encoding with the number 1 if the material was present in the structure and 0 if it was absent. After training, the decision tree had an accuracy of 0.86 on five-fold cross-validation. We chose entropy as a splitting criterion because the goal was to determine what parameter had the primary influence on the splitting and led to more information gain. Figure 6 shows the obtained decision tree. Geometry and choice of metals have a major impact on an emissivity curve profile. The decision tree algorithm firstly branches on the geometry, checking if the design has 2D periodicity on the surface. This split contains a significant information gain towards clusterization as the entropy value is decreased by one-third. With 2D periodicity (following the right arrow), the presence of tantalum or tungsten leads to the split into classes 4, 5, and 7. Class 6 is present all over the tree, so we neglect it from the consideration here. Following the left arrow, the tree goes deeper into geometry details, checking if the surface is flat or has grating. Class 1 mainly has "bull's eye" surface grating, while classes 2 and 3 are films with flat surfaces. This analysis corroborates the idea that geometry plays first.

10 Nine electronic paper scrapers

To check the presence of the keywords in figure captions, we implemented nine electronic paper scrapers for nine publishers. We were working with HTML versions of full-text papers by means of regular expressions. First, we manually determined unique HTML sequences used by each publisher to encode figure object and figure caption, we list them in Table 2. We chose HTML sequences in the way that the part of electronic paper from the start-sequence to the end-sequence contained only the caption sentence and other HTML encoding tags. Next, in every HTML paper, we located all start and end sequences with regular expressions and matched them in pairs combining start-sequence with the first following end-sequence. Then, we extracted paper parts for every start-end pair. As none of the HTML encoding tags contains "emissivity", "emitter" or "emission", we used regular expressions to check these paper parts for the presence of the keywords without any further processing. The algorithm took 2 days to run on the database of 4.9 million papers obtained through special publisher agreement.

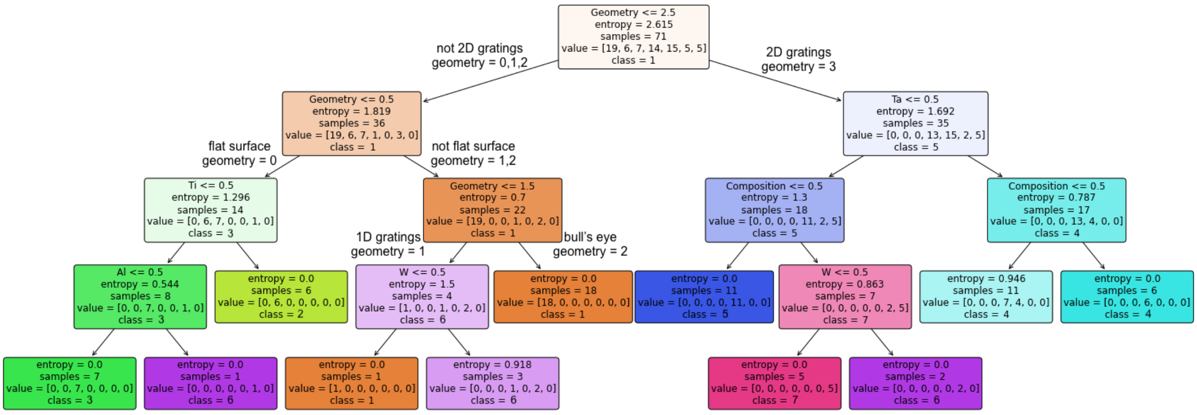

Figure 6: Decision tree plotted from metadata for the obtained classification. True arrow is on the left, splitting criterion is entropy representing the information gain. Geometry follow the numeric encoding: 0 - film, 1 - 1D grating, 2 - bull’s eye, 3 - 2D grating. Composition has binary encoding: 0 - single material, 1 - sandwich. For the material list, one-hot encoding is used.

PUBLISHER	HTML SEQUENCE INDICATING FIGURE CAPTION	
	START	END
Elsevier	<ce:caption	</ce:caption>
The Royal Society of Chemistry	<td class=”image_title	</td>
Wiley	<figcaption class	</figcaption>
American Institute of Physics	<span class= ”captionLabel”>FIG	<input id= ”showFigureAttributes
Nature Publishing Group	<figure	</figure>
American Chemical Society	<fig fig-type=”figure	</fig>
Springer	<figcaption	</figcaption>
American Physical Society	<label>FIG	</fig>
The Electrochemical Society	<span class=”fig-label”>	<div class=”sb-div caption-clear”>

Table 2: HTML sequences indicating figure captions for nine publishers.

References

1. *Lawrence Berkeley National Lab Natural Language Processing* https://lbnlp.github.io/lbnlp/. 2018.
2. Tshitoyan, V. *et al.* Unsupervised word embeddings capture latent knowledge from materials science literature. *Nature* **571,** 95–98 (July 2019).
3. Nadeau, D. & Sekine, S. A survey of named entity recognition and classification. *Lingvisticae Investigationes* **30,** 3–26 (2007).
4. Hochreiter, S. & Schmidhuber, J. Long short-term memory. *Neural computation* **9,** 1735–1780 (1997).
5. Bradski, G. The OpenCV Library. *Dr. Dobb’s Journal of Software Tools* (2000).
6. Canny, J. A computational approach to edge detection. *IEEE Transactions on pattern analysis and machine intelligence,* 679–698 (1986).
7. Matas, J., Galambos, C. & Kittler, J. Robust detection of lines using the progressive probabilistic hough transform. *Computer vision and image understanding* **78,** 119–137 (2000).
8. *EasyOCR* https://github.com/jaidedai/easyocr. 2020.
9. Dhakar, L. *Color Thief* https://lokeshdhakar.com/projects/color-thief/. 2020.
10. Pedregosa, F. *et al.* Scikit-learn: Machine Learning in Python. *Journal of Machine Learning Research* **12,** 2825–2830 (2011).
11. Havidek, H. *Dominant Color Detection* https://pypi.org/project/dominant-color-detection/. 2020.
